# Medical Opinion and Movement

**Published:** 1907-04-20

**Authors:** 


### Medical Opinion and Movement.

At a recent meeting of the Academy of Medicine in Paris, Dr. Vaillard, Director of the Military Medical School in Lyons, announced that he had discovered a specific serum for dysentery. This serum, which is obtained by the injection into horses of the bacillus in increasing doses, is specific for the so-called "dysenterie bacillaire," and the bacillus used is that discovered and isolated by Dr. Chantemesse. Dr. Vaillard, together with his son-in-law Dr. Dopter, has been working with the serum for the past year, and has treated in all 243 cases. He claims to have obtained remarkable successes. Out of these 243 cases, 99 were severe and 25 had been given up as hopeless. Fifteen out of these 25 were cured. Most of the experiments have been carried out at the Pasteur Institute, and the serum is now prepared there. Large doses can be given repeatedly without any ill effects. The author lays stress on the importance of injections in the early stage of the disease in order to obtain a rapid and certain cure. His results are corroborated by Dr. Chauvel, Dr. Vincent, and Dr. Widal. It is, of course, not likely that the serum will prove to be of any value for the treatment of the amœbic form of dysentery, such as occurs for the most part in tropical countries.

The Commission appointed to investigate the cause of Malta fever is to be congratulated on the work it has accomplished as a result of its researches during the last two or three years in Malta. It discovered that the disease is very prevalent among goats, and that they excrete the specific micrococcus in large numbers in their milk. In a paper read before the Epidemiological Society, Colonel Bruce gave an account of the work of the Commission, and showed that the facts afford overwhelming proof that infected goat's milk is the chief mode of infection for man. In consequence of this knowledge, measures have been taken to prevent the use of goat's milk, and, since it has been altogether excluded from the Royal Naval Hospital at Malta, not a single case of Malta fever has occurred there. Colonel Bruce has every hope that the disease will now entirely disappear from the garrison, and in consequence some 70,000 or 80,000 days of severe illness will be blotted out from the yearly medical reports of the army and navy. This will be an achievement of which the Commission may justly be proud, and for which no small amount of credit will be due to Colonel Bruce, who was the prime mover in the appointment of the Commission.

More than ten years ago deputations from the London County Council attended upon the Lord Chancellor to urge the necessity for changes in the methods of death certification and an amendment of the existing Coroners Acts, without, however, any practical result. Recently a further joint deputation was received by the Lord Chancellor from the Council and the Medico-Legal Society, headed by Sir William Collins and Mr. H. T. Anstruther, and from the sympathetic reply which it received there seems reason to hope that steps may be taken to alter the present unsatisfactory condition of affairs. Sir William Collins pointed out that the revelations made by the Death Certification Committee of 1893 exposed a public scandal, which has nevertheless remained unredressed for 14 years. The percentage of certified to total deaths varies from 1.2 per cent. in London to 8.5 in North Wales, and to 42 per cent. in Inverness-shire! The most important suggestions made by the deputation are: That no human body should be buried or otherwise disposed of without a certificate by a competent medical man or by a coroner; that the medical certificate should show the fact of death as well as its cause, and also vouch for the identity of the deceased; and that specially qualified and independent medical men should be appointed to assist the coroner in preliminary inquiries, in scrutinising the causes of death and in post-mortem examinations. There can be no question of the desirability of such technical advisers, especially in view of the fact that a large proportion of coroners are without any medical training, but proper provision should be made to secure also the assistance of the medical attendant of the deceased at all inquests and post-mortem examinations, and duly to remunerate him as heretofore. In many cases the medical attendant is in possession of facts which are essential to the proper elucidation of the cause of death, and his evidence is of importance not only at the inquests, but also in the preliminary inquiries and post-mortem examination.

One of the latest forms of treatment for that terrible malady, tic douloureux, consists in the injection of alcohol in the course of the affected nerve. It was originally devised by Dr. Schlosser, and appears to afford better results than the osmic acid injections. Dr. Oswalt of Paris has adopted a method of injection which he claims to be an improvement on the technique of Schlosser. He passes the needle within the mouth, behind the wisdom tooth, and slides it up inside the pterygoid plate until he reaches the opening of the foramen ovale. In this way he is able to inject the alcohol into the ganglion itself, and, further, by slightly altering the position of the needle, he can inject also into all three branches of the nerve. The method requires a very exact knowledge of the anatomy of the part, and must call for considerable judgment, which can only be acquired by practice, in order to direct the needle so that the point of injection exactly corresponds to the ganglion or the course of the individual branches of the nerve. On this account it is not a form of treatment which every general practitioner can easily undertake. Moreover, when a patient is suffering from a severe attack of the disease, he does not readily submit to the manipulation necessary to carry out the injection. This difficulty, of course, might be overcome by the administration of an anæsthetic, though Dr. Oswalt does not appear to have found this necessary.

